# Transcatheter and surgical aortic valve replacement for aortic stenosis in France: Trends from 2010 to 2022 and impact of European guidelines and clinical trial results

**DOI:** 10.1371/journal.pone.0351466

**Published:** 2026-06-16

**Authors:** Anaïs Havet, Annaëlle Testud, Aubane L’Hospital, Guy De Gevigney, Nicolas Chavanis, Marie Viprey, Xavier Armoiry

**Affiliations:** 1 Research on Healthcare Performance (RESHAPE), INSERM U1290, Université Claude Bernard Lyon 1, Lyon, France; 2 Hospices Civils de Lyon, Health Data Department, Lyon, France; 3 Department of Cardiology, Hôpital Louis-Pradel, Hospices Civils de Lyon, Lyon, France; 4 Department of Cardiac Surgery, Centre Médico-Chirurgical Léon Blum, Villeurbanne, France; 5 Pharmacy Department, Institut des sciences pharmaceutiques et biologiques (ISPB), Université Claude Bernard Lyon 1, UMR CNRS 5510 MATEIS, Edouard Herriot Hospital, Lyon, France; East Tennessee State University, UNITED STATES OF AMERICA

## Abstract

**Background:**

The PARTNER trials played a key role in the expansion of transcatheter aortic valve replacement (TAVR) for severe aortic valve stenosis (AS), shaping international practice guidelines. We assessed the evolution of AS management in France between 2010 and 2022, and the impact of PARTNER trials and European guideline updates on TAVR use. Characteristics of patients, facilities and TAVR valves, and post-procedure events were also described.

**Methods and findings:**

We conducted a nationwide cohort study using the French Health Data System (SNDS), including patients aged 18 years or older hospitalized for AS and receiving a first TAVR or surgical aortic valve replacement (SAVR) from 2010 to 2022. ARIMA models were used to study the impact of PARTNER trials and European guideline updates on TAVR use. Among 255,453 procedures, 109,739 were TAVR (N_2010_: 1,389; N_2022_: 16,770). No significant change in the proportion of TAVR was associated with PARTNER trials or European guideline updates (p ≥ 0.125). Median age and EuroSCORE II proxy were 83.0 years and 2.2 in TAVR group and 72.0 years and 1.4 in SAVR group. Approximately 60% of the TAVR and SAVR procedures were performed in public facilities. Latest valve generations replaced progressively earlier ones, with 62.5% being balloon-expandable. Mortality decreased over time in both groups, while length of stay and intensive care unit admissions decreased only in TAVR group.

**Conclusions:**

This 13-year nationwide overview highlights the growing uptake of TAVR in France, likely driven by clinical practice and procedural innovation rather than guidelines. Further analyses will compare efficacy and safety between TAVR and SAVR.

## Introduction

Aortic valve stenosis (AS) is one of the most prevalent forms of valvular heart disease in Europe. In France, hospitalizations for AS increased by 59% between 2006 and 2016, reaching 38.7 per 100,000 person-years in 2016 [1]. Its prevalence increases with age, affecting approximately 7% of individuals aged 75 and older [2].

Transcatheter aortic valve replacement (TAVR), developed by a French cardiologist and first performed in 2002, marked the beginning of a major change in the AS treatment. This minimally invasive procedure can be offered to patients for whom surgery is contraindicated and is now used regardless of surgical risk, with clinical benefits similar to those after surgical aortic valve replacement (SAVR) [3,4]. Several clinical trials, including the PARTNER trials, have compared TAVR and SAVR in terms of clinical outcomes and complications: PARTNER 1A (2010) enrolled high-risk surgical patients, while PARTNER 2 (2016) and PARTNER 3 (2019) included intermediate- and low-risk patients, respectively [4,5]. These trials reported positive findings and supported the progressive expansion of TAVR indications. They also stimulated the commercialization of a new generation of TAVR valves and delivery devices in order to improve clinical and safety outcomes. In parallel, the European Society of Cardiology (ESC) has progressively updated its guidelines on the management of severe AS, taking into account both surgical risk and patient age. In addition to non-operable and high-risk patients, the 2017 ESC guidelines expanded the use of TAVR to patients with intermediate surgical risk, after review by heart teams. Meanwhile, SAVR remained the recommended option for low-risk patients [6]. Based on the 2021 ESC guidelines, TAVR is now recommended for patients aged 75 and older, regardless of surgical risk, while SAVR is preferred for patients under 75 with low surgical risk [7].

Despite notable differences between the European and the American guidelines regarding the threshold of age and/or life expectancy for determining indications for TAVR and SAVR, the American College of Cardiology (ACC)/American Heart Association (AHA) guidelines have also validated the expansion of TAVR indications in their last updates [8,9].

To the best of our knowledge, no research work has been conducted to evaluate the impact of these trials and guidelines on TAVR use in real-world practice.

Here, we aimed to describe the evolution of AS management in France between 2010 and 2022, and to assess the impact of the PARTNER 2 and 3 trial results and the 2017 and 2021 ESC guideline updates on TAVR use. Additionally, we described the characteristics of treated patients, healthcare facility types, TAVR valves characteristics, and morbidity and mortality following the initial hospitalization.

## Materials and methods

### Study design and data source

We conducted a population-based cohort study using data from the National Health Data System (*Système National des Données de Santé*, or SNDS), a database containing information on all healthcare expenditures reimbursed by the national health insurance system. The SNDS covers 99% of the French population, and includes demographic data, chronic medical conditions (International Classification of Disease, 10^th^ version [ICD-10- codes]), and reimbursement data for outpatient care (physician or paramedical visits and procedures, drugs and medical devices dispensed, and lab tests) and inpatient care (diagnoses coded with ICD-10, date and duration of hospitalization and procedures) [10]. We also conducted a time series study to evaluate the impact of the PARTNER 2 (April 2016) and PARTNER 3 (May 2019) trial results, as well as the ESC guideline updates (August 2017 and August 2021), on TAVR use. We chose to focus on the PARTNER trials because they are the primary reference studies that established the efficacy and safety of TAVR and played a key role in its adoption in clinical practice. However, the impact of the PARTNER 1 trial, published in 2010, was not analysed because SNDS data prior to 2010 were deemed insufficiently complete or reliable.

The study was strictly observational and based on anonymized French medico-administrative data from the SNDS. In accordance with the French regulations governing non-interventional clinical research and the French Data Protection Supervisory Authority (CNIL), neither ethics committee approval nor written informed consent from participants was required. The research group has permanent access to these data under a specific regulatory framework.

### Study population

We included all patients aged 18 years or older who were hospitalized for heart failure, rheumatic or nonrheumatic aortic valve disease and/or congenital aortic valve failure (ICD-10 codes: I50, I06, I350, I352, Q231) and who had a first hospitalization for TAVR or SAVR between January 1, 2010, and December 31, 2022. The initial hospitalization for each patient was considered as the index stay. Patients were followed up from their index stay until January 31, 2023 or until they died, whichever came first. The following hospital stays were excluded: i) those occurring in healthcare facilities not authorised to perform TAVR procedures, ii) day-case stays, iii) stays for patients with inconsistent data on health insurance identifier, sociodemographic information or death, iv) stays for patients having a TAVR or SAVR for at least 4 years preceding the index stay, v) stays without diagnoses related to aortic stenosis, and vi) stays with both TAVR and SAVR (S1 Fig).

### Variables

The data on patient characteristics included sex, age, the number of comorbidities according to the Charlson index and the French Deprivation Index (FDep). The FDep is a socioeconomic measure used to assess the level of deprivation in the patient’s area of residence. It is derived from the 2015 census data. The FDep is categorized into quintiles, with the fifth quintile (Q5) representing patients living in the most deprived areas [11]. To approximate patients’ surgical risk, we developed a proxy of the EuroSCORE II using patient-, cardiac-, and surgery-related factors, identifiable in the SNDS [12] (S1 Table). Each patient’s score was calculated using the logistic regression coefficients from the official EuroSCORE II calculator. The resulting proxy score was then categorized into tertiles based on its distribution in the study population over the entire study period.

Healthcare facilities were categorized as public or private. Public facilities included university hospital centres and hospital centres, while private facilities included for-profit and not-for-profit private facilities.

We described the generation of TAVR valves (S2 Table), their type (self-expanding or balloon-expandable valves), and the name of manufacturers, including Medtronic, Edwards, Boston and Abbott.

For the analyses of morbidity and mortality, we evaluated length of stay and all-cause mortality rates at 30 days, 180 days and 1 year after the initial procedure. Within 30 days of the procedure, we also described the occurrence of complications: admission to the intensive care unit, myocardial infarction, stroke, pacemaker implantation and pulmonary embolism and the initiation of dialysis among individuals with no history of dialysis in the previous 12 months. Surgical reintervention and the time to first surgical reintervention were also described. Surgical reintervention was defined as a new hospital admission with a new TAVR and/or SAVR procedure.

### Statistical analyses

The number of TAVR and SAVR was plotted by year and age group (<65 years, 65–69 years, 70–74 years and ≥75 years) over the period 2010–2022. To account for changes in age structure over time, we also standardized the number of TAVR and SAVR to the 2023 French population aged 18 and over, using age-specific population data from the French National Institute of Statistics and Economic Studies (Insee) [13]. Additionally, out of all TAVR and SAVR procedures, we plotted the monthly percentage of TAVR from 2010 to 2022 and the monthly percentage of TAVR from 2013 to 2022 stratified by type of valve, due to a large amount of missing data on valve type prior to 2013. We also produced maps to visualize the percentage of TAVR (out of all TAVR and SAVR procedures) by department (a French administrative division), according to three time periods (2010–2015, 2016–2018 and 2019–2022). These periods were defined according to the publication dates of the PARTNER trials.

To assess the impact of the PARTNER 2 and 3 trial results and the 2017 and 2021 ESC guideline updates on monthly percentages of TAVR (out of all TAVR or SAVR procedures), over the period 2010–2022, we used autoregressive integrated moving average (ARIMA) models. These models included ramp functions (gradual changes) and step functions (immediate changes) in the time series [14]. Model fit was examined using autocorrelation plots, the augmented Dickey–Fuller test and Ljung-Box chi-square test. The optimal model was selected using the auto.arima function from the forecast package in R, based on the Akaike information criterion. Results are presented as estimated changes with 95% confidence intervals (95% CIs) and p values. We then applied similar models to evaluate i) the proportion of TAVR procedures performed with balloon-expandable valves (2013–2022), given their exclusive use in the PARTNER trials; ii) the percentage of patients aged 75 and older who underwent TAVR (out of all TAVR and SAVR procedures), to assess the impact of the 2021 ESC guideline update; and iii) changes in the mean EuroSCORE II proxy, to assess the effect of the PARTNER 2 and 3 trial results and the 2017 and 2021 ESC guideline updates.

The characteristics of patients, the type of healthcare facility and the morbidity and mortality following the index stay were described according to the type of procedure (TAVR or SAVR). For the analysis of morbidity and mortality following the initial procedure, we described the mean length of index stay, and within 30 days, admission to the intensive care unit (during index stay or a subsequent hospital stay), as well as the occurrence of complications. We also assessed all-cause mortality rates at 30 days, 180 days, and 1 year after the initial procedure, as well as surgical reintervention up to the end of the follow-up (31 January 2023), and the time to first reintervention. The occurrence of pacemaker implantation was further stratified by valve type because this procedure is more common with self-expanding valves than with balloon-expandable valves [15]. These variables as well as the type of TAVR valves were summarized using numbers and percentages for categorical variables, and the mean, standard deviation, median and interquartile range for continuous variable, according to three periods (2010–2015, 2016–2018 and 2019–2022).

## Results

### Evolution of aortic stenosis management and impact of the PARTNER trial results and the 2017 and 2021 ESC guideline updates on TAVR use

**Fig 1:**
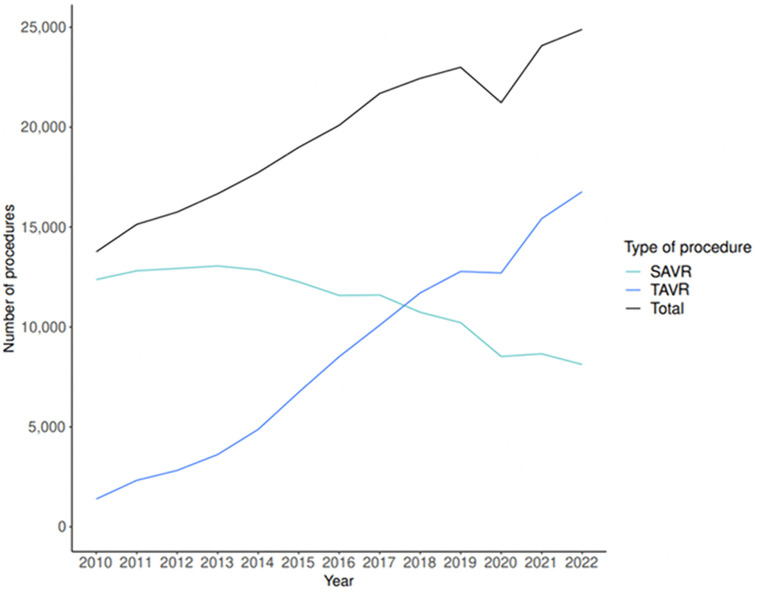
Type of AS management in France between 2010-2022. TAVR: Transcatheter Aortic Valve Replacement; SAVR: Surgical Aortic Valve Replacement..

**Fig 2:**
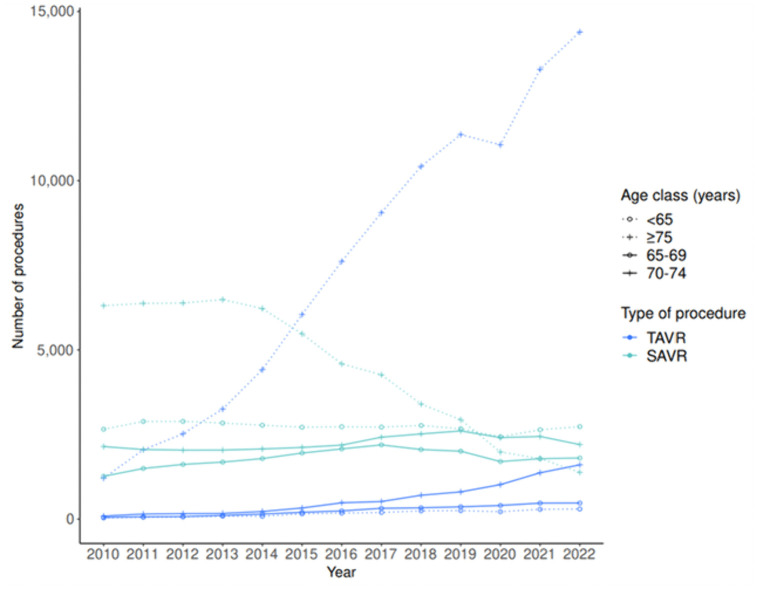
Evolution of AS management between 2010-2022 by age class. TAVR: Transcatheter Aortic Valve Replacement; SAVR: Surgical Aortic Valve Replacement.

Between January 1, 2010 and December 31, 2022, 255,453 TAVR and SAVR were identified, including 109,739 TAVR (43.0%) (S1 Fig). The annual number of procedures (TAVR and SAVR) increased by 80.0%, from 13,762 in 2010–24,893 in 2022. This upward trend was mainly associated with the increase in the number of TAVR, rising from 1,389 in 2010–16,770 in 2022. The number of SAVR remained stable between 2010 and 2015 with an average of 12,713 procedures per year, then gradually decreased from 11,579 surgeries in 2016–8,123 in 2022 (Fig 1). After age standardization, these trends remained similar (S2 Fig). We then stratified the analyses by age group and found that these trends were only observed among patients aged 75 years and older (Fig 2).

**Fig 3:**
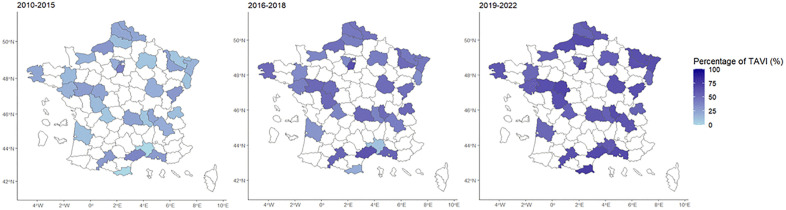
TAVR use by French departments between 2010-2022. The white areas correspond to areas in which healthcare facilities were not authorised to perform TAVR procedures. TAVR: Transcatheter Aortic Valve Replacement. Basemap: data.gouv.fr, distributed under the Open Licence Etalab 2.0.

Over the study period, the percentage of TAVR among all stays for aortic valve replacement increased across all French departments with healthcare facilities authorised to perform TAVR procedures. While disparities in practices were observed between departments during the 2010–2015 period, these differences appear to decrease by the 2019–2022 period. The proportion of TAVR among all procedures ranged from 10.1% to 35.4% during the 2010–2015 period, from 42.4% to 52.2% during 2016–2018 and from 55.6% to 67.4% during 2019–2022 (Fig 3).

**Fig 4:**
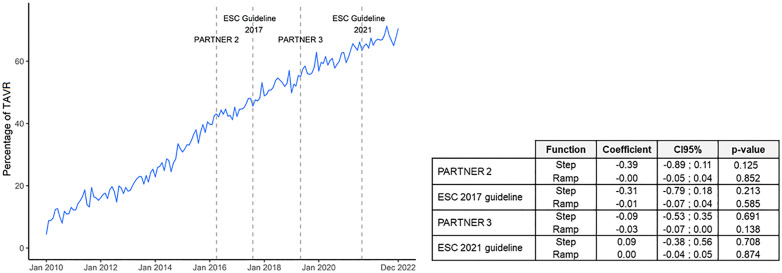
Impact of guidelines and PARTNER results on the TAVR percentage. TAVR: Transcatheter Aortic Valve Replacement; ESC: European Society of Cardiology; CI95%: 95% confidence interval.

**Fig 5:**
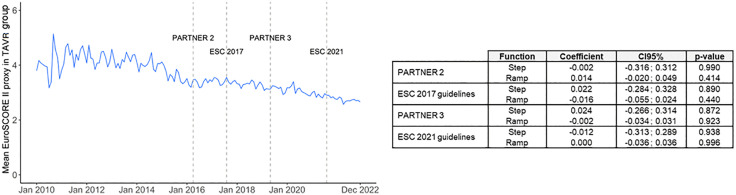
Impact of guidelines and PARTNER results on the mean EuroSCORE. TAVR: Transcatheter Aortic Valve Replacement; ESC: European Society of Cardiology; CI95%: 95% confidence interval.

The publication of the PARTNER trial results and that of the 2017 and 2021 ESC guideline updates were not significantly associated with an immediate or trend change in the percentage of TAVR among all procedures (p ≥ 0.125) (Fig 4). The publication of the PARTNER 2 trial results (TAVR for intermediate-risk patients) was associated with a slight upward trend in the percentage of TAVR performed with balloon-expandable valve (ramp = 0.28%) on all TAVR (p = 0.047) (S3 Fig). In other words, this publication was followed by a significant increase of 0.28% in the use of balloon-expandable valves. The PARTNER 3 trial results (TAVR for low-risk patients) were not significantly associated with an immediate or trend change in this percentage (p ≥ 0.746) (S3 Fig). No significant change in the percentage of TAVR among all TAVR or SAVR performed in patients aged 75 and older undergoing TAVR was observed following the 2021 ESC guideline updates (TAVR for patients ≥75 years old) (p ≥ 0.597, S4 Fig). Finally, the PARTNER trial results and the 2017 and 2021 ESC guideline update were also not significantly associated with changes on the mean proxy EuroSCORE (p ≥ 0.414) (Fig 5).

### Characteristics of patient and type of healthcare facility

Between 2010 and 2022, the median age of patients who underwent TAVR ranged from 84.0 years between 2010 and 2015 to 83.0 years between 2019 and 2022 and from 74.0 years between 2010 and 2015 to 69.0 years between 2019 and 2022 in SAVR group. Both TAVR and SAVR patients were mainly male (51.5% and 66.6%, respectively). Between 2010 and 2022, 21.4% of the TAVR group and 21.9% of the SAVR group lived in the most deprived areas (Q5 of FDep). The mean of number of comorbidities, according to the Charlson index, remained stable between 2010 and 2022 at 3.9 (±1.7) in TAVR patients, and at 3.2 (±1.6) in the SAVR group. The median proxy EuroSCORE II decreased over time, from 2.8 between 2010 and 2015 to 2.0 between 2019 and 2022 in the TAVR group, and from 1.5 to 1.2 in the SAVR group during the same periods. The percentage of TAVR patients with a proxy EuroSCORE II ≤ 1.6 was 23.0% between 2010 and 2015, and increased to 38.9% between 2019 and 2022. A similar, but less marked, increase was observed in the SAVR group, from 55.0% to 62.3% over the same periods. Furthermore, over the 2010–2022 period, the distribution of patients according to the EuroSCORE II proxy was balanced across the three risk categories in the TAVR group, whereas in the SAVR group, the majority of patients were classified in the low-risk category (Table 1).

**Table 1:** Patient characteristics by procedure type (TAVR or SAVR) between 2010 and 2022.

	2010 - 2015		2016-2018		2019-2022		Total period 2010–2022	
	TAVR (N = 21,759)	SAVR (N = 76,279)	TAVR (N = 30,307)	SAVR (N = 33,915)	TAVR (N = 57,673)	SAVR (N = 35,520)	TAVR (N = 109,739)	SAVR (N = 145,714)
Age, mean (±SD)	83.0 (6.9)	71.8 (10.7)	82.8 (6.8)	69.8 (10.0)	81.9 (6.9)	67.8 (9.3)	82.4 (6.9)	70.4 (10.4)
Age, median [q1, q3]	84.0 [80.0, 88.0]	74.0 [66.0, 80.0]	84.0 [80.0, 87.0]	71.0 [65.0, 77.0]	83.0 [78.0, 87.0]	69.0 [63.0, 74.0]	83.0 [79.0, 87.0]	72.0 [65.0, 78.0]
Men, N (%)	10,653 (49.0)	48,541 (63.6)	15,047 (49.6)	23,009 (67.8)	30,834 (53.5)	25,441 (71.6)	56,534 (51.5)	96,991 (66.6)
FDep, N (%)								
Q1	3,913 (19.1)	11,177 (15.5)	5,516 (18.6)	5,257 (15.9)	9,999 (17.7)	5,442 (15.8)	19,428 (18.2)	21,876 (15.7)
Q2	3,862 (18.8)	13,543 (18.8)	5,480 (18.4)	6,110 (18.5)	10,213 (18.1)	6,516 (18.9)	19,555 (18.3)	26,169 (18.7)
Q3	4,079 (19.9)	14,818 (20.5)	5,868 (19.7)	6,793 (20.6)	11,526 (20.4)	7,103 (20.6)	21,473 (20.1)	28,714 (20.6)
Q4	4,351 (21.2)	16,742 (23.2)	6,486 (21.8)	7,600 (23.0)	12,598 (22.3)	8,033 (23.3)	23,435 (22.0)	32,375 (23.2)
Q5	4,313 (21.0)	15,877 (22.0)	6,366 (21.4)	7,280 (22.0)	12,146 (21.5)	7,421 (21.5)	22,825 (21.4)	30,578 (21.9)Nu
Unknown	1,241	4,122	591	875	1,191	1,005	3,023	6,002
Number of comorbidities according to the Charlson index								
Mean (±SD)	4.1 (1.7)	3.2 (1.6)	3.9 (1.7)	3.2 (1.6)	3.8 (1.7)	3.2 (1.6)	3.9 (1.7)	3.2 (1.6)
Median [q1,q3]	4.0 [3.0, 5.0]	3.0 [2.0, 4.0]	4.0 [3.0, 5.0]	3.0 [2.0, 4.0]	4.0 [3.0, 5.0]	3.0 [2.0, 4.0]	4.0 [3.0, 5.0]	3.0 [2.0, 4.0]
EuroSCORE II proxy								
Mean (±SD)	4.0 (3.7)	2.6 (3.3)	3.4 (3.0)	2.4 (3.2)	2.9 (2.7)	2.3 (3.1)	3.3 (3.1)	2.5 (3.2)
Median [q1, q3]	2.8 [1.7, 4.96]	1.5 [1.0, 2.8]	2.3 [1.5, 4.2]	1.3 [0.9, 2.6]	2.0 [1.3, 3.7]	1.2 [0.8, 2.5]	2.2 [1.4, 4.1]	1.4 [0.9, 2.7]
EuroSCORE II proxy, N (%)^a^								
Low (≤1.6)	4,999 (23.0)	41,928 (55.0)	9,418 (31.1)	20,572 (60.7)	22,419 (38.9)	22,138 (62.3)	36,836 (33.6)	84,638 (58.1)
Intermediate (>1.6 and ≤3.4)	7,532 (34.6)	18,880 (24.8)	10,270 (33.9)	6,895 (20.3)	18,699 (32.4)	6,992 (19.7)	36,501 (33.3)	32,767 (22.5)
High (>3.4)	9,228 (42.4)	15,471 (20.3)	10,619 (35.0)	6,448 (19.0)	16,555 (28.7)	6,390 (18.0)	36,402 (33.2)	28,309 (19.4)

TAVR: Transcatheter Aortic Valve Replacement; SAVR: Surgical Aortic Valve Replacement; SD: Standard Deviation; q: quartile; Q: Quintile; FDep: French Deprivation index; ^a^Calculated on the basis of pseudo EuroSCORE II tertiles for the entire population over the period 2010–2022.

Between 2010 and 2022, 42.3% of TAVR were performed in private facilities and 57.7% in public facilities, and 39.6% of SAVR were performed in private facilities and 60.4% in public facilities (S3 Table).

### Characteristics of TAVR valves

Between 2010 and 2022, as technical advances led to the marketing of newer valve generations, first- and second-generation transcatheter aortic valves were gradually replaced by third- and fourth-generation devices. A total of 62.5% of TAVR valves were balloon-expandable and 37.5% were self-expanding. Edwards balloon-expandable valves and Medtronic self-expanding valves were almost exclusively used in France during this period, with market shares of 62.5% and 34.4% respectively (Table 2).

**Table 2:** Generation and type of TAVR valve and name of manufacturer, from 2010 to 2022.

	2010-2015 (N = 21,759)	2016-2018 (N = 30,307)	2019-2022 (N = 57,673)	Period 2010–2022 (N = 109,739)
Generation of TAVR, N (%)				
First generation	5,460 (25.1)	615 (2.0)	354 (0.6)	6,429 (5.9)
Second generation	5,044 (23.2)	9,448 (31.2)	8,194 (14.2)	22,686 (20.7)
Third generation	675 (3.1)	3,545 (11.7)	12,438 (21.6)	16,659 (15.2)
Fourth generation	4,583 (21.1)	15,956 (52.6)	35,750 (62.0)	56,289 (51.3)
Missing data	5,997 (27.6)	743 (2.5)	937 (1.6)	7,677 (7.0)
Type of TAVR, N (%)				
Self-expandable	5,493 (25.2)	11,699 (38.6)	21,084 (36.6)	38,276 (34.9)
Balloon expandable	10,269 (47.2)	17,865 (58.9)	35,652 (61.8)	63,786 (58.1)
Missing data	5,997 (27.6)	743 (2.4)	937 (1.6)	7,677 (7.0)
Manufacturer, N (%)				
Medtronic	5,493 (25.2)	11,699 (38.6)	17,915 (31.1)	35,107 (32.0)
Edwards	10,269 (47.2)	17,865 (58.9)	35,652 (61.8)	63,786 (58.1)
Abbott	0.0 (0.0)	0.0 (0.0)	1,117 (1.9)	1,117 (1.0)
Boston	0.0 (0.0)	0.0 (0.0)	2,052 (3.6)	2,052 (1.9)
Missing data	5,997 (27.6)	743 (2.5)	937 (1.6)	7,677 (7.0)

TAVR: Transcatheter Aortic Valve Replacement.

### Morbidity and mortality after valve implantation

#### TAVR group.

Between 2010 and 2022, the mean length of stay was 8.2 days (±7.2) in TAVR group, decreasing from 11.2 days (±8.8) between 2010 and 2015 to 6.8 days (±6.0) between 2019 and 2022. A total of 15% of TAVR procedures were followed by an admission to intensive care unit within 30 days between 2010 and 2015, compared to 6.1% between 2019 and 2022. Within 30 days following TAVR, the percentages of myocardial infarction, stroke, pulmonary embolism or dialysis were 0.2%, 0.9%, 0.1% and 0.1% respectively, and remained stable over the 2010–2022 period. The percentage of pacemaker implantations within 30 days following TAVR increased from 16.6% between 2010 and 2015 to 18.3% between 2019 and 2022. Pacemaker implantation within 30 days occurred in 22.1% of cases after a self-expanding valve and in 15.7% after a balloon-expandable valve over the period 2010–2022. Between the periods 2010–2015 and 2019–2022, the 30-day mortality rate declined from 5.4% to 2.2%, the 180-day mortality rate from 11.1% to 6.8%, and the 1-year mortality rate from 15.6% to 12.4%. Between 2010 and 2022, the percentage of surgical reinterventions following TAVR remained low (≤0.4%), with a mean time to first reintervention of 934.6 days (±1,007.9), corresponding approximately 2.5 years (±2.8) (Table 3).

**Table 3:** Morbidity and mortality following the initial TAVR procedure, by period from 2010 to 2022.

	2010-2015	2016-2018	2019-2022	Period 2010–2022
	(N = 21,759)	(N = 30,307)	(N = 57,673)	(N = 109,739)
Length of stay, mean (±SD)	11.2 (8.8)	8.7 (7.3)	6.8 (6.0)	8.2 (7.2)
Within 30 days following the first TAVR procedure, N (%):				
Admission to intensive care unit^1^	3,259 (15.0)	2,635 (8.7)	3,520 (6.1)	9,414 (8.6)
Myocardial infarction^a^	52 (0.2)	82 (0.3)	138 (0.2)	272 (0.2)
Stroke^a^	171 (0.8)	232 (0.8)	518 (0.9)	921 (0.9)
Pacemaker implantation^a^	3,619 (16.6)	5,510 (18.2)	10,527 (18.3)	19,656 (17.9)
after self-expanding valve^b^	1,330 (24.2)	2,635 (22.5)	4,486 (21.3)	8,451 (22.1)
after balloon-expandable valve^c^	1,387 (13.5)	2,732 (15.3)	5,876 (16.5)	9,995 (15.7)
Pulmonary embolism^1^	22 (0.1)	33 (0.1)	39 (0.1)	94 (0.1)
Dialysis^d^	50 (0.2)	41 (0.1)	53 (0.1)	144 (0.1)
Death, N (%)^e^				
at 30 days	1,182 (5.4)	886 (2.9)	1,291 (2.2)	3,359 (3.1)
at 180 days	2,414 (11.1)	2,171 (7.2)	3,498 (6.8)	8,083 (7.8)
At 1 year	3,389 (15.6)	3,482 (11.5)	5,372 (12.4)	12,243 (12.9)
Surgical reintervention, N (%)^f^				
with TAVR	229 (1.1)	119 (0.4)	135 (0.2)	483 (0.4)
with SAVR	86 (0.4)	98 (0.3)	114 (0.2)	298 (0.3)
with TAVR/SAVR^g^	N < 10	N < 10	N < 10	N < 10
Time to first surgical reintervention, mean (±SD)	1,547.3 (1171.7)	801.0 (722.3)	281.6 (295.8)	934.6 (1007.9)

SD: standard deviation; ^a^Among patients with ≥30 days of follow-up, or with complications within 30 days following the procedure, representing 99.9% of the population; ^b^N_2010-2022_ = 38,277 patients with pacemaker implantation after self-expanding valve; ^c^N_2010-2022_ = 63,786 patients with pacemaker implantation after balloon-expandable valve; ^d^Described among patients without a history of dialysis in the year prior to the initial procedure (N_2010-2022_ = 107,528); ^e^Described among patients with at least 30 days (N_2010-2022_ = 109,712), 180 days (N_2010-2022_ = 103,537), 1 year (N_2010-2022_ = 95,268) of follow-up after the TAVR procedure; ^f^Described until the end of patient follow-up (no later than January 31, 2023); ^g^TAVR and SAVR procedures coded on the same day.

#### SAVR group.

Between 2010 and 2022, the mean length of stay remained relatively stable in SAVR group, with 13.9 days (±10.2). The percentage of admission to intensive care within 30 days following SAVR was high and increased over the study period, rising from 46.4% between 2010 and 2015 to 53.4% between 2019 and 2022. Within 30 days following SAVR, the percentages of myocardial infarction, stroke, pulmonary embolism or dialysis were 0.5%, 0.4%, 0.1% and 0.2% respectively, and remained stable over the 2010–2022 period. The percentage of pacemaker implantations within 30 days following SAVR was stable over time, at 5.4% between 2010 and 2022. Between the periods 2010–2015 and 2019–2022, the 30-day mortality rate declined from 2.8% to 2.1%, the 180-day mortality rate from 4.7% to 3.9%, and the 1-year mortality rate from 5.9% to 5.4%. Between 2010 and 2022, following SAVR, the percentage of surgical reintervention by TAVR or SAVR was 1.5% and 2.2%, respectively. The mean time to first reintervention was 1,867.7 days (±1,184.1), corresponding to approximately 5.1 years (±3.2) (Table 4).

**Table 4:** Morbidity and mortality following the initial SAVR procedure, by period from 2010 to 2022.

	2010-2015	2016-2018	2019-2022	Period 2010–2022
	N = 76,279	N = 33,915	N = 35,520	N = 145,714
Length of stay, mean (±SD)	14.3 (10.7)	13.5 (9.8)	13.3 (9.5)	13.9 (10.2)
Within 30 days following the first SAVR procedure, N (%):				
Admission to intensive care unit^a^	35,377 (46.4)	16,744 (49.4)	18,974 (53.4)	71,095 (48.8)
Myocardial infarction^a^	328 (0.4)	147 (0.4)	200 (0.6)	675 (0.5)
Stroke^a^	285 (0.4)	121 (0.4)	151 (0.4)	557 (0.4)
Pacemaker implantation^a^	4,031 (5.3)	1,937 (5.7)	1,832 (5.2)	7,800 (5.4)
Pulmonary embolism^a^	42 (0.1)	16 (0.0)	15 (0.0)	73 (0.1)
Dialysis^b^	139 (0.2)	63 (0.2)	49 (0.1)	251 (0.2)
Death, N (%)^c^				
at 30 days	2,129 (2.8)	799 (2.4)	749 (2.1)	3,677 (2.5)
at 180 days	3,575 (4.7)	1,284 (3.8)	1,258 (3.9)	6,117 (4.3)
At 1 year	4,488 (5.9)	1,637 (4.8)	1,534 (5.4)	7,659 (5.5)
Surgical reintervention, N (%)^d^				
with TAVR	1,806 (2.4)	283 (0.8)	37 (0.1)	2,126 (1.5)
with SAVR	2,186 (2.9)	685 (2.0)	348 (1.0)	3,219 (2.2)
with TAVR/SAVR^e^	1 (0.0)	1 (0.0)	0 (0.0)	2 (0.0)
Time to first surgical reintervention, mean (±SD)	2,188.4 (1,141.8)	1 111.7 (713.4)	444.3 (373.7)	1 867.7 (1,184.1)

SD: standard deviation; ^a^Among patients with ≥30 days of follow-up, or with complications within 30 days following the procedure, representing 99.9% of the population; ^b^Described among patients without a history of dialysis in the year prior to the initial procedure (N_2010-2022_ = 144,313); ^c^Described among patients with at least 30 days (N_2010-2022_ = 145,695), 180 days (N_2010-2022_ = 142,698), 1 year (N_2010-2022_ = 138,581) of follow-up after the SAVR procedure; ^d^Described until the end of patient follow-up (no later than January 31, 2023); ^e^TAVR and SAVR procedures coded on the same day.

## Discussion

The study aimed to evaluate the evolution of AS patient management in France between 2010 and 2022, as well as the impact of the PARTNER trial and the ESC guideline updates on TAVR use. To this end, we conducted a nationwide study using the potential from the French health insurance claims database. Over a thirteen-year observation period, we identified over 255,000 patients, of whom nearly 110,000 underwent TAVR. To the best of our knowledge, this is one of the largest cohort studies conducted in Europe.

We showed that the number of TAVR procedures has risen twelvefold from 2010 to 2022 in France, and this increase has not been offset by a proportional decrease in SAVR procedures. A transient decrease in the number of procedures observed around 2020 may reflect the impact of the COVID-19 pandemic, which disrupted elective surgical activity in France. These findings suggest that the expansion of TAVR indications enables the management of a wider range of patients with AS and the continued use of SAVR in specific indications, particularly in younger patients, or in those with concomitant valve disease or coronary artery disease [4,7]. These trends have also been reported in previously published French studies based on registry and hospital data with more limited cohort size and period of follow-up [1,16–18]. TAVR is now widely accepted by the medical community and available in 65 countries worldwide, with an annual compound growth rate of 40% [19].

Here, the first ARIMA model showed no immediate or trend change in the proportion of patients who underwent TAVR among all procedures, at the time of publication of the PARTNER trial results and the ESC guideline updates. However, this does not necessarily imply that the trials results and guidelines had no influence. Rather, it suggests that the adoption of TAVR was already underway prior to their publication, driven by the combined effect of the growing scientific evidence, increasing number of authorised centres in France, accumulating clinical experience and technological improvements. The publication of the trials and the updated guidelines may have reinforced this ongoing dynamic, but without producing a sudden shift detectable by time-series methods [17,20–22]. In addition, given the strong pre-existing upward trend, the ARIMA model may have had limited sensitivity to detect discrete changes in level or slope around specific events, which should be considered when interpreting these findings.

Similarly, the publication of the PARTNER trial results was not associated with any immediate or trend change in the percentage of TAVR performed with a balloon-expandable valve. However, here again, we cannot conclude that these trials had no impact, as the use of these valves was already increasing before the publication, and the increase was maintained throughout the study period. Unexpectedly, we also observed a drop in the use of balloon-expandable valves between September and November of 2016. This drop likely the result of the recall issued by the French Medicines Agency (ANSM) in September 2016 concerning Edwards Lifesciences’ batch withdrawal of the Edwards Commander® valve delivery system, following modifications to the catheter body manufacturing process.

The mean age of patients undergoing TAVR or SAVR decreased slightly over time. This is presumably due to the expansion of TAVR indication to lower-risk patients, as well as the latest ESC guidelines recommending SAVR for younger patients under 75 years of age [7]. Furthermore, the median age of patients who underwent TAVR was higher than that of SAVR, as TAVR was recommended in frail, older patients with a higher surgical risk [4]. The mean Euroscore II proxy decreased in both TAVR and SAVR groups over the years and was lower in the SAVR group, which is consistent with the latest ESC guidelines [4,7].

The findings regarding TAVR valve characteristics align with those of international and French studies, which report the widespread and steadily increasing use of Edwards Sapien® valves [17,23]. In fact, the use of these balloon-expandable valves was found to be associated with a lower risk of aortic regurgitation and pacemaker implantation at 30 days compared with self-expanding valves, which may partly explain their wider adoption [24]. Furthermore, the higher proportion of pacemaker implantation within 30 days after self-expanding valves compared with balloon-expandable valves was consistent with previously reported data [15]. Notably, pacemaker implantation rates increased over time across both valve types, which may reflect changes in anatomical characteristics, implantation techniques, and evolving clinical practices [25,26].

Mortality rate at 30-days decreased over time in both groups and this finding was consistent with the results reported in studies based on PMSI data and national registries [1,27]. One of these studies also estimated TAVR- and SAVR-related mortality using medical causes of death, reporting 30-day mortality rates of 3.4% for TAVR and 2.7% for SAVR, and one-year rates of 11.3% and 5.2%, respectively [1]. A study based on data from the France 2 registry reported low rates of myocardial infarction within 30 days and one year following TAVR [28]. The same study also showed a low one-year stroke rate after TAVR, but a higher 30-day stroke rate compared with our study (2.3% versus 0.9%). This difference in 30-day stroke rates may be due to differences in the stroke identification algorithms, which were not thoroughly explained in the previous publication.

### Strengths and limitations

Most of previous French studies on the use of TAVR or SAVR were mainly based on hospital data up to 2019 or registry data [18,29,30], and only one study also included outpatient data, limited to the period 2006–2016 [1]. The present study used more recent SNDS data, covering both hospital and outpatient care. With a 13-year study period, it provides a comprehensive and detailed overview of surgical practices, including the adoption of latest-generation valves.

The severity of AS is not recorded in the SNDS, which means we cannot ensure that all included patients had severe AS. Furthermore, the indication of procedures is not available in this database. We therefore decided to include patients hospitalized for heart failure, since aortic valve stenosis can progress to heart failure and cause its symptoms. These patients accounted for 1.4% of the study population, and their inclusion is unlikely to have influenced the overall results. Conversely, patients hospitalized for aortic insufficiency were not included, as TAVR is not indicated in this condition. TAVR requires valve calcifications for fixation, which are absent in aortic insufficiency. Additionally, patients who underwent both TAVR and SAVR during the same stay were also excluded, as it was impossible to determine the order of the procedures. However, these cases represented less than 0.2% of the total.

In this study, an EuroSCORE II proxy was developed in collaboration with a cardiologist and a cardiac surgeon, including not only hospital data as in previous studies, but also outpatient care data, such as drug reimbursements and biological and medical procedures [12,31]. However, it was challenging to identify some of this score’s criteria using SNDS data alone due to the lack of clinical data. This may have introduced classification bias and led to an underestimation of patients’ surgical risk. Despite this limitation, our EuroSCORE II proxy allowed us to describe changes in patients’ surgical risk over time and provide a more specific assessment of cardiac surgical risk than the number of comorbidities derived from the Charlson index.

The SNDS database does not specify the underlying reasons for reintervention, making it difficult to distinguish those related to complications from those due to prosthetic degeneration. Early reinterventions may reflect technical issues such as paravalvular leak, whereas late reinterventions are more often linked to prosthetic degeneration. In addition, reintervention results should be interpreted with caution, as follow-up duration differed across the study periods, with shorter follow-up in more recent cohorts potentially leading to an underestimation of reintervention proportions. Lastly, due to the descriptive nature of this study, a direct comparison of morbidity and mortality between SAVR and TAVR procedures was not performed. However, a comparative analysis is planned using high-dimensional propensity scores proxy, which have demonstrated good performance in controlling for unmeasured confounding by indication in previous comparative effectiveness studies [32].

Finally, although TAVR adoption is increasing worldwide and American guidelines have also expanded indications to patients with lower surgical risk, our study evaluated the impact of European guidelines in France. Differences in healthcare organisation and clinical context between countries should be taken into account, and caution is warranted when generalising these findings beyond France.

## Conclusions

This study provides a 13-year overview of AS management in France, highlighting the growing accessibility and uptake of TAVR nationwide. This proactive adoption by heart teams appears to have been driven by clinical experience, technical innovation, and the expansion of authorised centres, with guidelines likely reinforcing these emerging practices.

Improvements in morbidity and mortality following initial TAVR procedures are consistent with the expansion of indications to lower-risk patients, as well as with technical advances in TAVR. For health policymakers, this study emphasized the importance of real-world data for monitoring the dissemination and outcomes of costly procedures such as TAVR. This initial work provides a thorough description of TAVR and SAVR practices in France. Additional analyses are underway to compare the efficacy and safety of these two procedures, taking into account patients’ surgical risk profiles based on the EuroSCORE proxy developed in this study.

## Supporting information

S1 Table EuroSCORE II items and corresponding SNDS variables. (DOCX)

S2 Table Generation and type of TAVR valves by manufacturer. (DOCX)

S3 Table Number of TAVR or SAVR by type of healthcare facility, across three periods. (DOCX)

S1 Fig Selection of the study population. (DOCX)

S2 Fig Age-standardized number of TAVR and SAVR procedures by year, from 2010 to 2022. (DOCX)

S3 Fig Evolution of the percentage of TAVR by type of valve and impact of the PARTNER 2 and 3 trial results on the percentage of balloon-expandable valves (n = 210,803). (DOCX)

S4 Fig Impact of the ESC2021 guideline update on the proportion of patients aged 75 and older. (DOCX)
