# Supplementary material for: Transcatheter and surgical aortic valve replacement for aortic stenosis in France: Trends from 2010 to 2022 and impact of European guidelines and clinical trial results
Source: PLoS One. 2026 Jun 16;21(6):e0351466. doi: 10.1371/journal.pone.0351466 (PMC13271474; doi:10.1371/journal.pone.0351466)
Supplement: S1 Table — (DOCX) [file pone.0351466.s001.docx]

**S1 Table. EuroSCORE II items and corresponding SNDS variables**

| **EuroSCORE II items** | **Corresponding SNDS variables** |
| --- | --- |
| **Patient related factors** | |
| Age | - |
| Sex | - |
| Chronic respiratory disease | bronchodilator use  *and/or* COPD hospitalization |
| Extracardiac arteriopathy | hospitalization for peripheral arterial disease and/or carotid artery stenosis/occlusion  *and/or* previous intervention for abdominal aortic and/or carotid |
| Poor mobility | purchase or rental of wheelchairs, canes/crutches, or walkers |
| Previous cardiac surgery | - |
| Active endocarditis | hospitalization for endocarditis  *and* dispensation of antibiotic treatment |
| Insulin-treated diabetes | - |
| Critical preoperative state | hospitalization for cardiac arrest and/or sudden death and/or acute renal failure  *and/or*  medical act for intra-aortic balloon pump implantation  *and/or*  admission to intensive care or critical care unit before the initial surgery |
| Chronic kidney disease | kidney failure with or without dialysis |
| **Cardiac related factors** | |
| Recent myocardial infarction (within 90 days) | - |
| Heart failure class (NYHA) | class II: no hospitalization for heart failure within the previous 12 months  class III: patients identified from the study population  class IV: patients identified from the study population with a hospitalization for pulmonary oedema or cardiogenic shock within the previous 12 months |
| Left ventricular ejection fraction | low LVEF: patients with cardiomyopathy  reduced LVEF: patients with heart failure |
| Angina at rest | - |
| **Surgery related factors** | |
| Urgent intervention | admission through the emergency department during the same stay |
| Thoracic aorta surgery | - |
| Operative weight | only applicable to SAVR procedures with associated CABG (Coronary Artery Bypass Grafting) or MAZE procedures  Weight:  1: SAVR procedure alone  2: SAVR procedure with one CABG or MAZE procedure  3: SAVR procedure with ≥2 CABG or MAZE procedures |
