# Supplementary material for: Transcatheter and surgical aortic valve replacement for aortic stenosis in France: Trends from 2010 to 2022 and impact of European guidelines and clinical trial results
Source: PLoS One. 2026 Jun 16;21(6):e0351466. doi: 10.1371/journal.pone.0351466 (PMC13271474; doi:10.1371/journal.pone.0351466)
Supplement: S2 Table — (DOCX) [file pone.0351466.s002.docx]

| **Manufacturer** | **Name** | **Generation** | **Type** | **LPP Code** |
| --- | --- | --- | --- | --- |
| Medtronic | Corevalve | 1^st^ | Self-expanding | 3269300  3235301 |
|  | Evolut | 1^st^ | Self-expanding | 3288467 |
|  | Evolut R | 2^nd^ | Self-expanding | 3267005 |
|  | Evolut Pro | 3^rd^ | Self-expanding | 3279860 |
|  | Evolut Pro + | 4^th^ | Self-expanding | 3288929 |
| Edwards | Sapiens | 1^st^ |  |  |
|  | Sapien XT | 2^nd^ | Balloon-expandable | 3239144  3205292  3241796 |
|  | Sapien 3, Certitude | 3^rd^ | Balloon-expandable | 3239865 |
|  | Sapien 3 Ultra, Commander | 4^th^ | Balloon-expandable | 3299070 |
| Abbott | PORTICO | 1^st^ | Self-expanding | 3279178 |
|  | PORTICO FLEXNAV | 2^nd^ | Self-expanding | 3245340 |
|  | NAVITOR | 3^rd^ | Self-expanding | 3238200 |
| Boston | LOTUS | 1^st^ | Self-expanding | 3200509 |
|  | LOTUS EDGE | 2^nd^ | Self-expanding | 3261103 |
|  | ACURATE NEO | 3^rd^ | Self-expanding | 3290501 |
| LPP: Liste des Produits et Prestations (French reimbursement code for medical devices) | | | | |

**S2 Table. Generation and type of TAVR valves by manufacturer**
