# Supplementary material for: Transcatheter and surgical aortic valve replacement for aortic stenosis in France: Trends from 2010 to 2022 and impact of European guidelines and clinical trial results
Source: PLoS One. 2026 Jun 16;21(6):e0351466. doi: 10.1371/journal.pone.0351466 (PMC13271474; doi:10.1371/journal.pone.0351466)
Supplement: S3 Table — (DOCX) [file pone.0351466.s003.docx]

**S3 Table. Number of TAVR or SAVR by type of healthcare facility, across three periods**

| N (%) | 2010-2015 | 2016-2018 | 2019-2022 | Period 2010-2022 |
| --- | --- | --- | --- | --- |
| TAVR | N=21,759 | N=30,307 | N=57,673 | N=109,739 |
| Private | 8,577 (39.4) | 12,826 (42.3) | 25,035 (43.4) | 46,438 (42.3) |
| Public | 13,182 (60.6) | 17,481 (57.7) | 32,638 (56.6) | 63,301 (57.7) |
| SAVR | N=76,279 | N=33,915 | N=35,520 | N=145,714 |
| Private | 29,860 (39.1) | 13,495 (39.8) | 14,332 (40.3) | 57,687 (39.6) |
| Public | 46,419 (60.9) | 20,420 (60.2) | 21,188 (59.7) | 88,027 (60.4) |
| TAVR: Transcatheter Aortic Valve Replacement; SAVR: Surgical Aortic Valve Replacement. | | | | |
