# Supplementary material for: Transcatheter and surgical aortic valve replacement for aortic stenosis in France: Trends from 2010 to 2022 and impact of European guidelines and clinical trial results
Source: PLoS One. 2026 Jun 16;21(6):e0351466. doi: 10.1371/journal.pone.0351466 (PMC13271474; doi:10.1371/journal.pone.0351466)
Supplement: S1 Fig — (DOCX) [file pone.0351466.s004.docx]

 **S1 Fig. Selection of the study population**
