# Supplementary material for: Transcatheter and surgical aortic valve replacement for aortic stenosis in France: Trends from 2010 to 2022 and impact of European guidelines and clinical trial results
Source: PLoS One. 2026 Jun 16;21(6):e0351466. doi: 10.1371/journal.pone.0351466 (PMC13271474; doi:10.1371/journal.pone.0351466)
Supplement: S3 Fig — (DOCX) [file pone.0351466.s006.docx]

|  | **Function** | **Coefficient** | **CI95%** | **p-value** |
| --- | --- | --- | --- | --- |
| PARTNER 2 | Step | 1.81 | -2.347 ; 5.971 | 0.393 |
|  | Ramp | 0.28 | 0.003 ; 0.566 | 0.047 |
| PARTNER 3 | Step | -0.107 | -4.199 ; 3.983 | 0.959 |
|  | Ramp | 0.078 | -0.396 ; 0.553 | 0.746 |

**
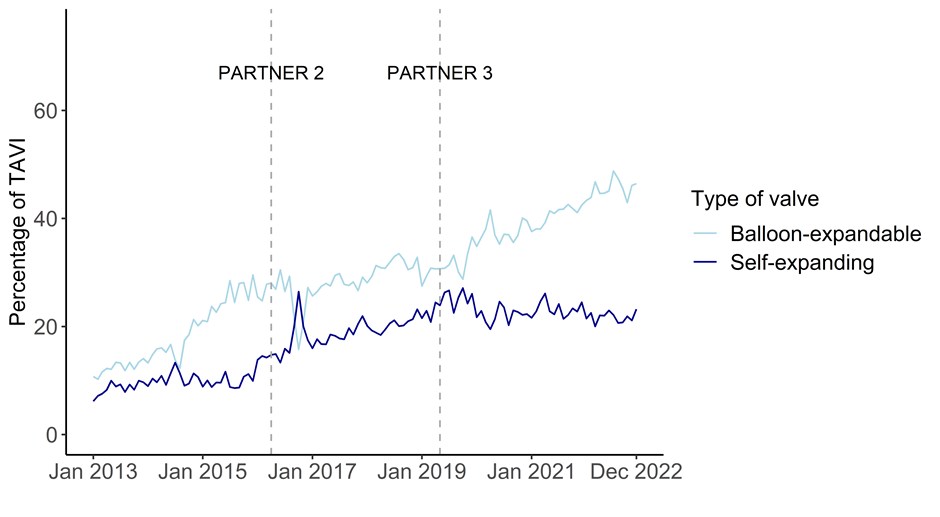
**


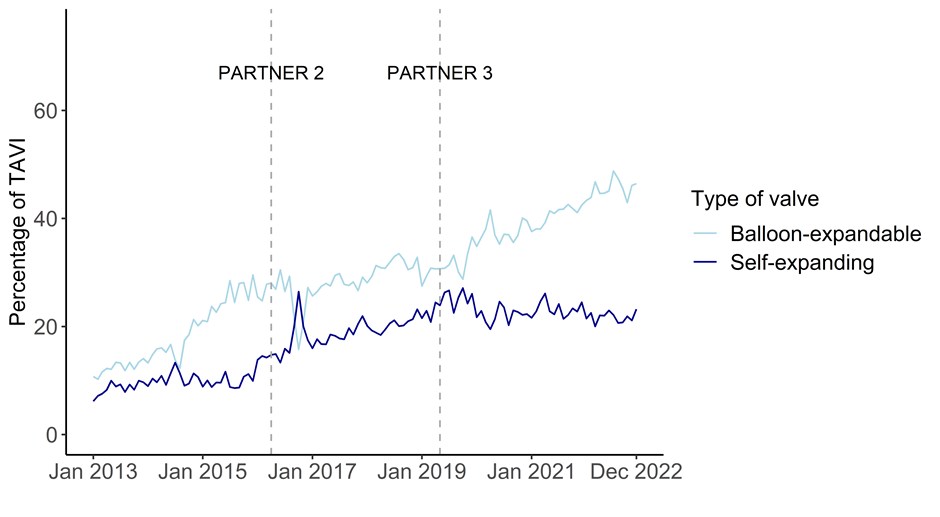

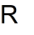


**S3 Fig. Evolution of the percentage of TAVR by type of valve and impact of the PARTNER 2 and 3 trial results on the percentage of balloon-expandable valves (n=210,803)**
