# Supplementary material for: Transcatheter and surgical aortic valve replacement for aortic stenosis in France: Trends from 2010 to 2022 and impact of European guidelines and clinical trial results
Source: PLoS One. 2026 Jun 16;21(6):e0351466. doi: 10.1371/journal.pone.0351466 (PMC13271474; doi:10.1371/journal.pone.0351466)
Supplement: S4 Fig — (DOCX) [file pone.0351466.s007.docx]

|  | **Function** | **Coefficient** | **CI95%** | **p-value** |
| --- | --- | --- | --- | --- |
| ESC 2021 guidelines | Step | -0.01 | -0.02 ; 0.01 | 0.597 |
|  | Ramp | -0.00 | -0.00 ; 0.00 | 0.762 |

**
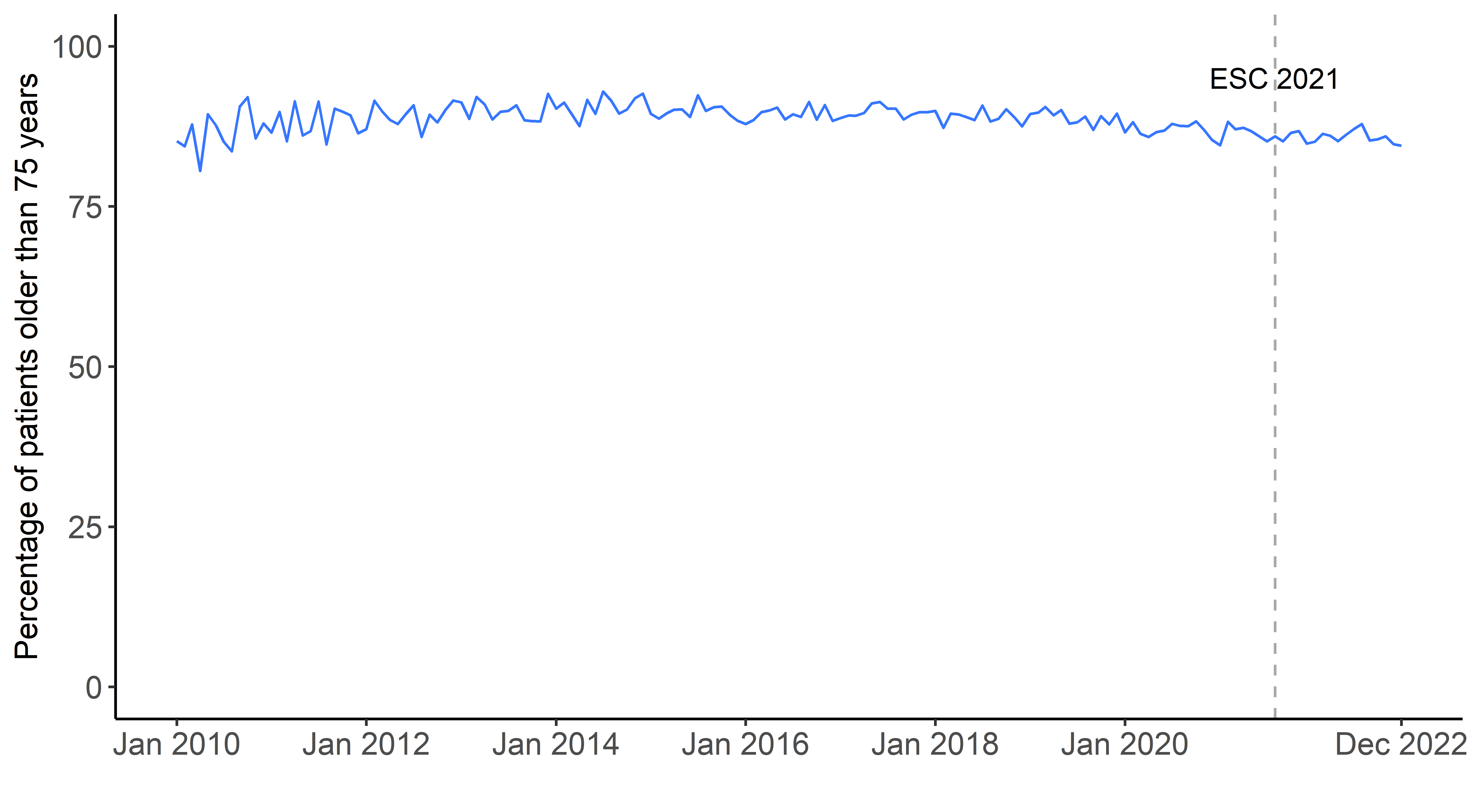
**

**S4 Fig. Impact of the ESC2021 guideline update on the proportion of patients aged 75 and older**
